# Present-Day Problems

**Published:** 1888-12-22

**Authors:** 


### THE DOCTOR'S PULPIT.

(*Continued from page* 164.)

### PRESENT-DAY PROBLEMS.

### CURES FOR "SOCIAL PARASITES."

"THE wise man's eyes are in his head," said Solomon. A policy of stupidity is the beginning of all misery. If a man must take a leap in the dark, he must. But no sane man will take a leap in the dark who can have daylight for his neck-risking plunge. Is there any man in London who does not feel the pressure of the rates? Is there, indeed, any man in Great Britain? Even a woman, before she has been married five years, learns to know the call of the collector, and to put the blue paper out of the way if her husband happens to return home in the evening tired or cross. The appeal is here made to self-interest, because the poorest intelligence can understand that. There are grounds far higher and worthier on which the question of the Cure of "Social Parasitism" might be argued. But only one person here and there has either intelligence or imagination enough to care for arguments on very high grounds. A corn bin a mile off may be a very feeble persuader for a horse, but a gadfly on his buttocks makes his tail fly about with no doubtful energy. The pauper gadflies, the hospital gadflies, and the begging gadflies are attacking the social quadruped, not only on the buttocks, but on every part of his unprotected skin. If the animal have any sense at all he will at least look at them, "take stock" of them, and consider whether or not anything can be done, and what, in order to get rid of them.

It was admitted last week that idlers, "inbred" paupers, and thieves ought to be made to suffer. Even if they suffer to the verge of starvation they are justly punished. But an intelligent community should be able to make its able-bodied idlers and its shiftless persons earn at least a part of their keep. It is a distinct confession of stupidity and want of governing power on the part of those in authority to say that they do not know what to do with workless people and with idlers and voluntary paupers. In medical practice, with which we are now running a parallel, the first thing the physician always tries to do is to establish his diagnosis. "What actually is the matter with the patient at this moment?" he asks himself. When that question is answered, clearly, distinctly, unmistakably, the cure can be usually set about with confidence and hope. Is it too much to ask the state doctor to proceed on similar lines? It is infallibly certain that nothing will be done in the way of real cure until some such lines are proceeded upon. It may be said the patients are so numerous that the task of dealing with them is impossible. Nothing of the kind! They are numerous, but they do not all suffer from different diseases. At most the different diseases are not more than half-a-dozen; all the patients may be divided into five or six classes, and a suitable treatment can then be adopted for each class. What sort of a physician would he be who should huddle together into one huge ward cases of scarlet fever, eczema, nettlerash, and chicken-pox, on the ground that they had all got an affection of the skin? Yet the deserving poor and the undeserving, the idle and the industrious, when they fall below a certain level, are all indiscriminately lumped together under the one common description of "paupers." What a coarse and stupid method of dealing with our fellow-soldiers in life's warfare! There is not even an approximation to the use of elementary scientific principles. With us a man is either an independent and free citizen, or he is a pauper, disgraced and discredited for ever.

Now, at least, this measure of classification is possible. A man is either a willing pauper or an unwilling. But therein lies a whole universe of difference. The willing pauper is human dross and refuse; the unwilling may be the purest human gold. To the willing pauper the money of the state is welcome as sunshine in winter; it is the very thing he wants. To the unwilling, every penny that touches his fingers is like a red-hot coal; he hates the sight of it; the mere presence is degradation. We will not ask, Is it fair, or is it just, or is it brotherly to put on the man who hates pauperism in his very soul, the same label and description as you put on the man whose heart loves nothing so well as idleness and other people's food? We will ask, Is it wise; will it pay; will it answer your purpose; is it "good business"? It is the worst possible business; it does not pay; it leads to present loss and ultimate crime. Have we yet to learn that in every essential element of character the worthy poor are exactly the same as the worthy rich? Their self-respect is as great, their pride as great, their sense of honour as worthy, their sense of shame as deep. In proportion to their numbers, the character of the poor may always be compared, without fear, with the character of the rich.

It is insisted then, first of all, that there are poor and poor, and that those in authority should be able to discriminate between the two classes; not only to discriminate, but to separate them; to separate them finally, and always, so that there shall be no kind of connexion whatever between the two; so that they shall never be named, or even thought of, as belonging to the same order. Suppose your human beings were houses, would you, because they had a few slates off the roof or a window or two broken, condemn them as useless houses and sell them for pulling down? But this is what is often done with poor men whom misfortune, and not vice, puts temporarily out of repair. Such men are ruthlessly thrust into the pauper ranks, with the certainty that they will find it very difficult to get out again, and quite impossible ever to wash away the pauper stain.

What, then, is to be done with the "involuntary pauper"? First recognise him, then remove him from the pauper class, then find him work. How the last is to be done will be considered later. If the first and second steps are taken, the initial stage of the cure of social parasitism may be considered accomplished. The paupers will then be a class who deserve the name. The able-bodied among them should be compelled to work at productive labour; the disabled may be left to such lighter duties as they are fit for. There is no reason whatever why able-bodied voluntary paupers should not be hired out by parishes to anyone who chooses to employ such labour; there is no reason why they should not be sent into the army or the navy; no reason why they should not be shipped to the colonies or to America or elsewhere, if a market can be found for their work. If no market can be found for them, then let them have such food and lodging as will keep body and soul together, and no more. If hardship be their lot, they must always remember that that lot is their own voluntary choice. But on behalf of the involuntary paupers—the poor whom misfortune has overtaken—self-interest as well as humanity speaks in different tones. It says: " These are men and women whom a little present help may shortly restore to the normal condition of independent, self-supporting citizens. The help they want is work."

Are we driven to make the shameful admission that the "resources of the Empire" are inadequate to the finding of occasional work for a few ten thousands of our deserving poor? We either can or we cannot find the work. To say that we cannot is to utter a lie which is as flagrant as it is contemptible. What! With our wide dominions, our unequalled revenue, and our unused wealth—we cannot find work for a hundred thousand pairs of willing hands! It may as well be said that summer heat cannot melt the icicles which gather about the window-pane in winter, or that the wind cannot blow a child's toy-ship half a mile from the shore. If it be said we will not, that it is everybody's business and nobody's business, and that therefore the thing is not done, that is credible and true. But to say that we cannot is to utter an absurdity as ridiculous as that the sun will rise in the west to-morrow morning, and set in the south. For shiftless persons, for inbred paupers, for incorrigible beggars, and for voluntary thieves, hardly any severity can be too severe. But for hands and hearts that are willing to work and to earn their own bread so long as strength shall last, for these science and religion, self-interest and humanity alike, demand that work be found by which they can live in health and wellbeing.

*(To be continued.)*

